# Heterotopy (*“Error loci”*) of the spiral loop of the ascending colon in cattle

**DOI:** 10.1371/journal.pone.0215402

**Published:** 2019-04-12

**Authors:** Arcangelo Gentile, Marilena Bolcato, Gianfranco Militerno, Günter Rademacher, André Desrochers, Annamaria Grandis

**Affiliations:** 1 Department of Veterinary Medical Sciences, University of Bologna, Bologna, Italy; 2 Clinic for Ruminants with Ambulatory and Herd Health Services at the Centre for Clinical Veterinary Medicine, LMU Munich, Munich, Germany; 3 Department of Clinical Science, Faculty of Veterinary Medicine, Université de Montréal, Saint-Hyacinthe, Canada; Universita degli Studi di Napoli Federico II, ITALY

## Abstract

The term heterotopy of the spiral colon encompasses a dysmorphological condition in which the spiral loops of the ascending colon (SLACs) do not form an orderly spiraling mass adjacent to the left side of the mesojejunum. As a consequence, the spiral loops are spread over a larger surface, making them more or less movable. It has been hypothesized that the abnormal position of the spiral loops of the ascending colon might constitute a predisposing factor for an intestinal obstruction or an ileus condition. The objective of the present study was to evaluate the anatomy of the spiral loops of the ascending colon in a population of healthy calves and to determine the prevalence of dysmorphism. The investigation was carried out on 1113 slaughtered veal calves. In 472 out of the 1113 calves, the spiral loops showed conformational aspects different from what has so far been described as normal in reference textbooks. In 91 calves the condition was definitely considered a pathological deviation from normality: in fact, the spiral colon had lost its typical spiral shape with random spacing between the loops, and it was nearly or completely detached from the mesojejunum. The lack of a broad attachment of the spiral loops of the ascending colon to the mesentery could provoke an alteration of the intestinal centre of gravity, enhancing the already asymmetrical distribution of weight between the jejunum and the descending colon.

## Introduction

In ruminants, the large colon (*Colon crassum*) is composed of the ascending colon (*Colon ascendens*), the transverse colon (*Colon transversum*) and the descending colon (*Colon descendens*). Furthermore, the ascending colon is divided into the proximal loop (*Ansa proximalis coli*), the spiral loop (*Ansa spiralis coli*) and the distal loop (*Ansa distalis coli*) [1]. The ascending colon is functionally associated with the caecum and is often associated with any caecal abnormalities. The spiral loop of the ascending colon (SLAC) is an elliptical coil on a single plane where there are 1.5–2 centripetal gyri, and the same number of centrifugal gyri with a central flexure between them [2–4]. Other than atresia coli, specific anomalies affecting the spiral loop are rare. Atresia coli is a lethal congenital disease if not treated. Affected calves are lacking parts or the entire spiral, and the distal loops of the ascending colon as well as the descending colon [5–7].

In 2008, Rademacher and Gentile [8] reported a repeated occurrence of intraoperatory and post-mortem findings of morphological distortions of the SLAC. The findings were mostly observed in animals affected by mechanical intestinal obstruction diseases, such as volvulus, torsion and intussusception. Those variations had not previously been described in the veterinary anatomical literature. They consisted of an irregular space between the loops and loss of the disc-shaped coil morphology with superimposed loops on a different plane. This dysmorphysm was defined as “heterotopy” of the spiral colon, this term encompassing all the conditions in which the loops of the spiral colon do not form an orderly single plane coil with its mesocolon fused to the left side of the mesojejunum. Therefore, it was hypothesised that the abnormal position of the SLAC, still to be verified as to whether it is congenital or acquired, might constitute a predisposing factor for intestinal obstruction or an ileus condition. The predisposition would be based on an impairment of the intestinal centre of gravity, and the consequently reduced capacity of the intestine, or tracts of it, of maintaining its correct anatomical position when subjected to stress causing abnormal movements of the intestinal mass [8]. In 2011, Wölfl [9] examined the morphology of 213 ascending colons; the study included non specifically-addressed intraoperative laparatomic observations and post-mortem evaluations. Only 129 out of the 213 SLACs (60.6%) had a normal morphology.

The objective of the present study was to evaluate the anatomy of the SLAC in a population of healthy calves and to determine the prevalence of dysmorphism.

## Materials and methods

The animals selected for this study were obtained from a single slaughterhouse in November 2010; 1113 Holstein veal male calves were examined post-mortem. Their origin was unknown whereas their diet, according to the Italian regulation for veal rearing, was expected to be based on milk replacer and a small amount of fibrous food (corn silage and/or grain). Specific age was not available but their slaughter weight (body weight ranging from 240 to 275 kg) put them between 6 to 8 months of age. They were healthy and without abnormalities at pre-slaughter clinical investigation. Previous medical history was not available.

**Fig 1:**
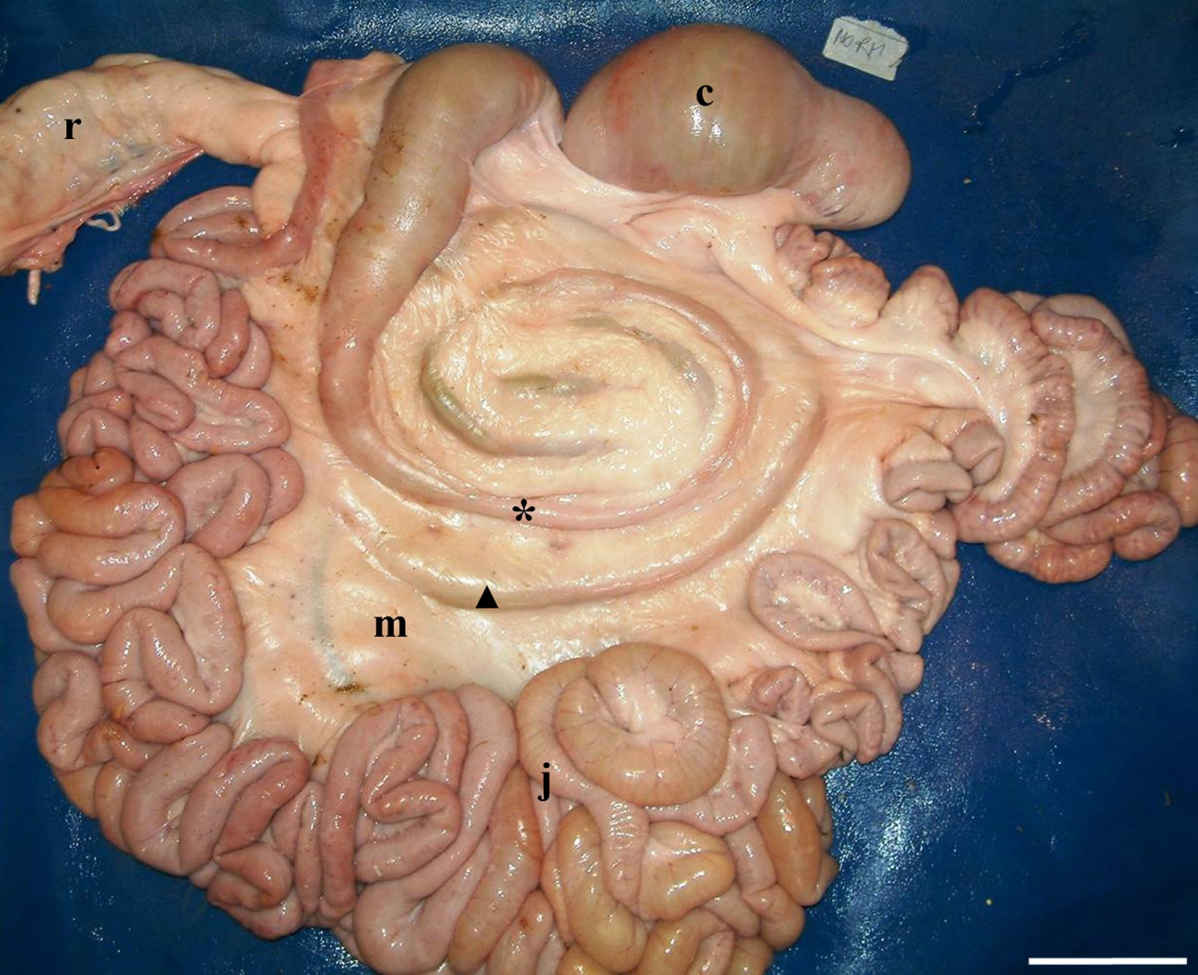
Normal topography of the SLAC, located at the middle of the mesojejunum, as reported in the reference textbooks. The rectum was displaced cranially to allow better visualisation of the Ansa proximalis coli. Notice the abundant presence of fatty tissue filling the mesentery plane. The asterisk indicates the first centripetal coil. The triangle indicates the last centrifugal coil. c: caecum; j: jejunum; m: mesojejunum; r: rectum. (Medial left side view of the intestine; bar = 10 cm).

**Fig 2:**
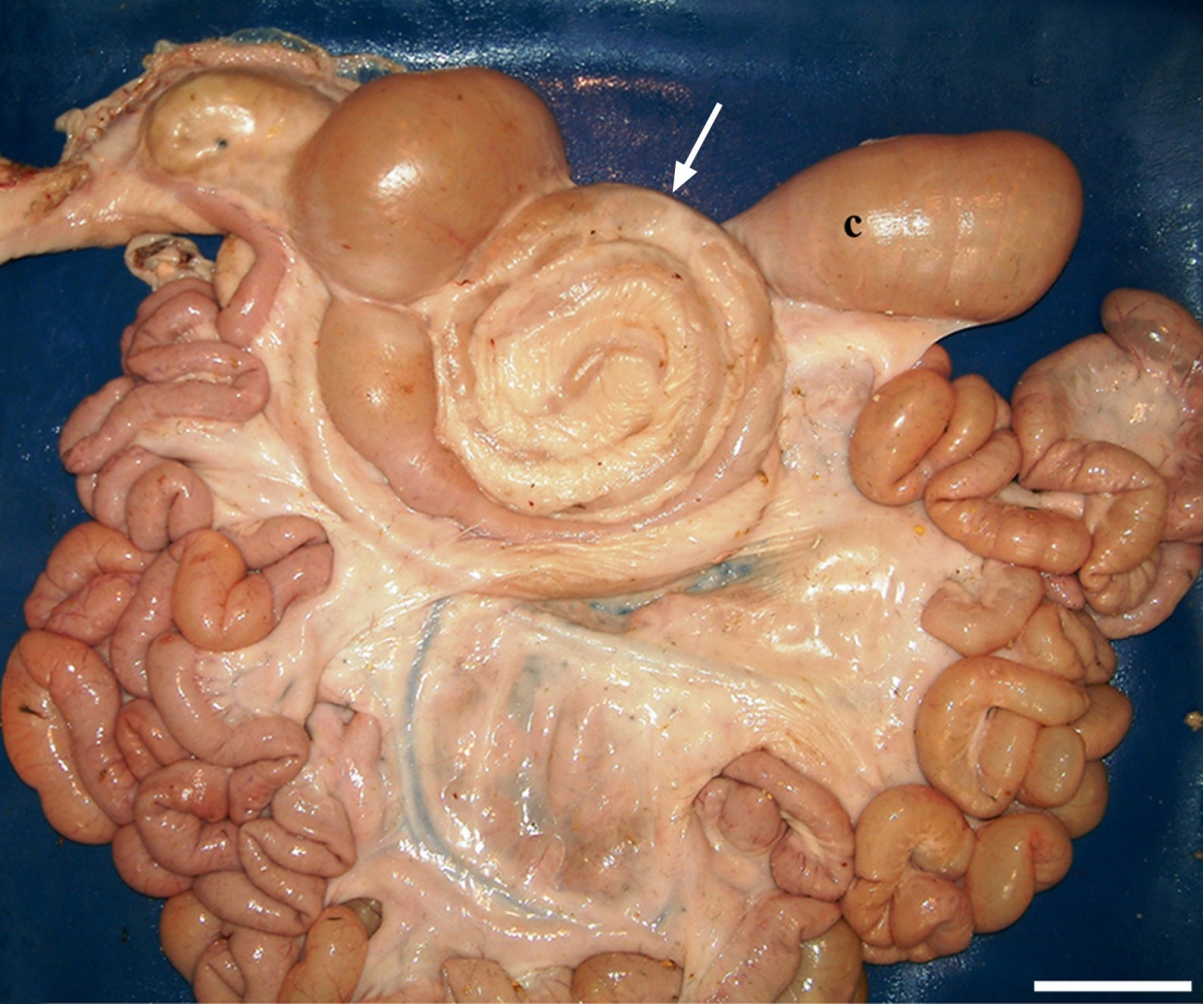
Conical shape looseness. The SLAC is so loosly attached to the mesojejunum that it can easily be displaced dorsally to overlap the last centrifugal coil (the most external loop; arrow) and the first portion of the caecum (c). (Medial left side view of the intestine; bars = 10 cm).

**Fig 3:**
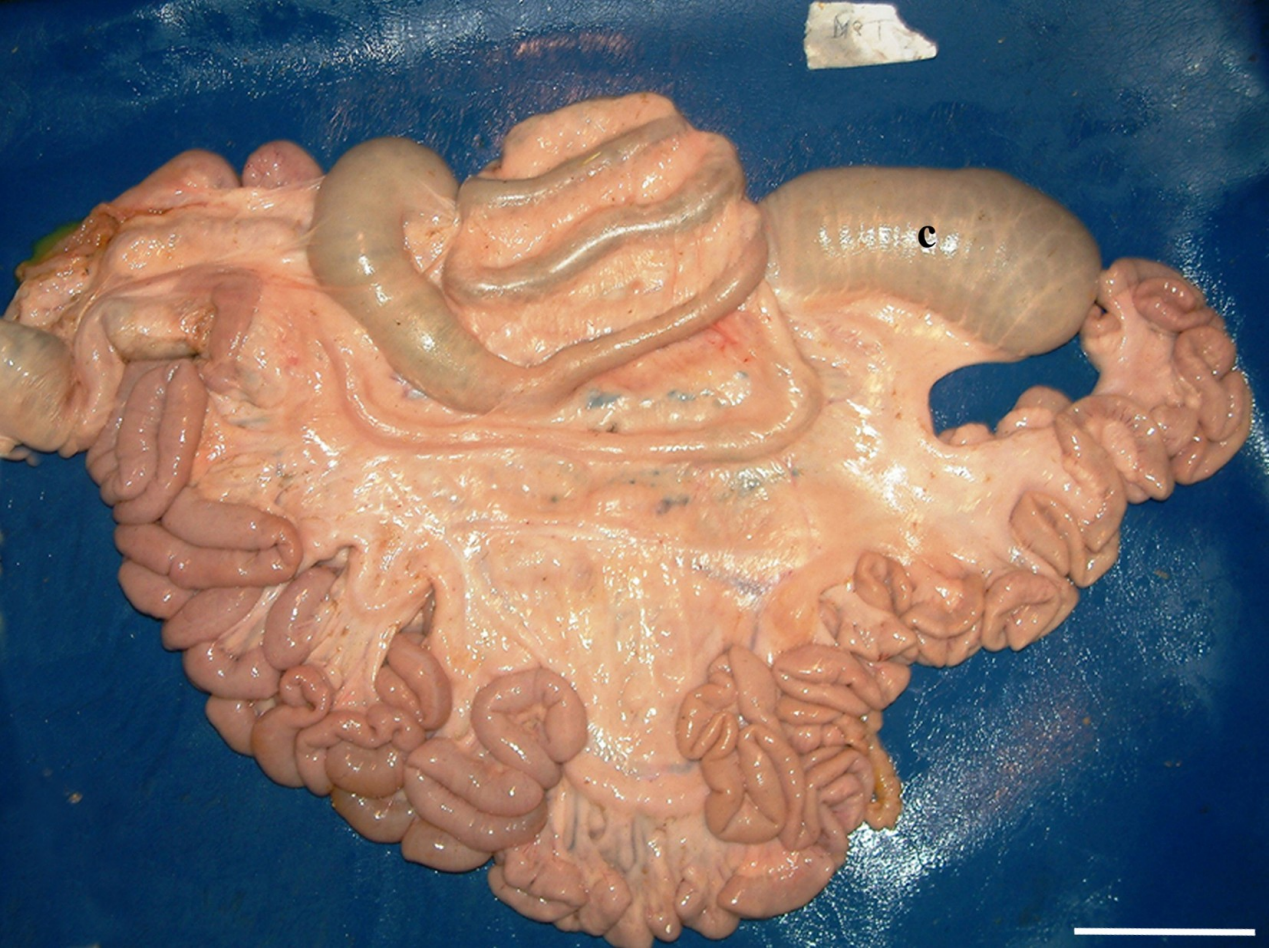
Partial dystopia. The SLAC is partially detached and displaced from the mesojejunum with an elongated mesocolon. The coiling is abnormal. c: caecum. (Medial left side view of the intestine; bar = 10 cm).

**Fig 4:**
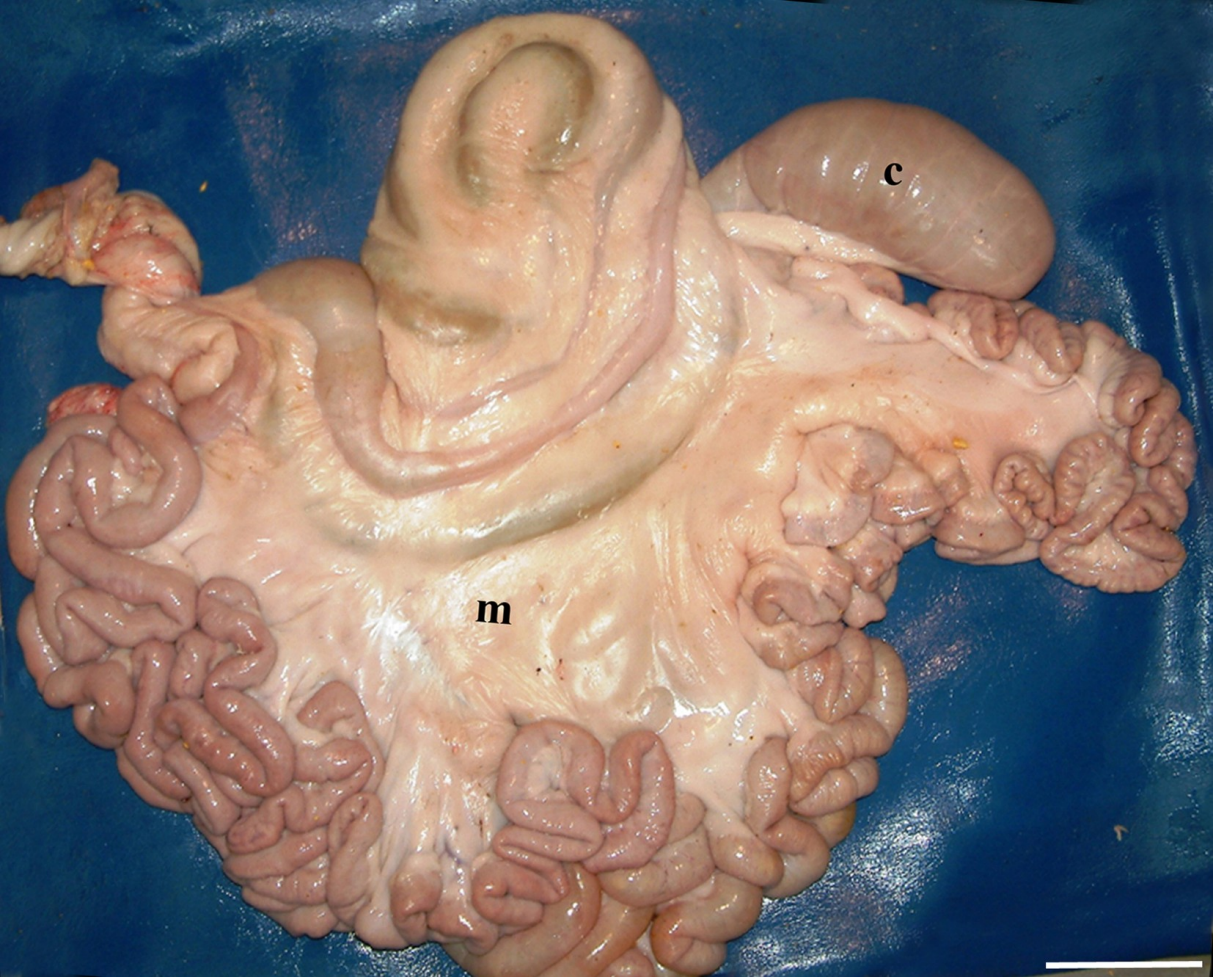
Complete ectopia. The SLAC is completely detached and completely displaced from the mesojejunum. The coiling is evidently abnormal. C: caecum; m: mesojejunum. (Medial left side view of the intestine; bar = 10 cm).

The intestinal tract was cut out by the employees of the slaughterhouse and put into a container for additional observation in the viscera processing room. The intestinal tract was always spread out the same way with its left side uppermost. The same observer carried out all the evaluations. The specific configuration of the SLAC was classified on the basis of the space between the loops and their abnormal superimposition as well as on the loss of the disc shape configuration:

1. equal distance between the loops to the mesojejunum: This referred to the normal anatomical situation in which the centripetal and the centrifugal coils of the SLAC were intimately attached to the left side of the mesojejunum, and the loops are ordenlty distended without any overlapping among adjacent loops (Fig 1);
2. conical shape looseness of the SLAC: The spiral loops are loosely attached to the mesojejunum and, if raised to the level of the central flexure they assume a tridimensional flaccid conical shape. Some loops can overlap adjacent loops (Fig 2);
3. partial dystopia [(dys- +‎ -topia, from ancient Greek δυσ- ‎(dus- = “bad”) + τόπος ‎(tópos = “place”)] of the SLAC: The intestinal segments of the SLAC are separated from each other with an elongated mesocolon. The overlapping of some intestinal segments is more significant (Fig 3);
4. complete ectopia [ektós- + -topia, from ancient Greek ἐκτός- ‎(ecto- = “outside”)] of the SLAC: In this case, the SLAC is completely detached from the mesojejunum. Therefore, it is “dislocated” from its position in the middle of the mesojejunum, as if it were an independent anatomical structure (Fig 4). Incomplete coiling can also be present.

The frequency of each configuration was expressed in a percentage with a 95% confidence interval.

## Results

The observations regarding the SLACs of the 1113 slaughtered calves are summarised in Table 1. A normal configuration, as expected according to the veterinary anatomical literature, was observed in 641 (57.6± 2.9%) out of the 1113 calves. A conical shape looseness of the SLAC was found in 381 out of 1113 (34.2±2.8%). Partial dystopia of the SLAC was noted in 68 out of 1113 cases (6.1±1.4%) whereas complete ectopia was present in 23 out of 1113 calves (2.1±0.8%).

**Table 1:** Distribution of the different aspects of the SLAC in 1113 slaughtered veal calves.

Aspects of the SLAC in 1,113 slaughtered veal calves	Number of observations	Frequency±confidence interval
Equal distance between the loops to the mesojejunum	641	57.6 ± 2.9%
Conical shape looseness of the SLAC	381	34.2±2.8%
Partial dystopia of the SLAC	68	6.1±1.4%
Complete ectopia of the SLAC	23	2.1±0.8%

## Discussion

This study clearly indicated that the heterotopy of the SLAC represented a relatively common anatomical anomaly in young clinically healthy animals of the Holstein breed. In fact, 42.4% of the SLACs examined in this study had significant anatomical divergence from what is normally described in reference textbooks. The anomalies observed went from loose attachment of the spiral loops to the mesojejunum, abnormal elongation of the mesocolon, overlapping of adjacent loops, abnormal coiling and finally to complete detachment of the SLAC from the mesojejunum. While conical shape looseness, observed in 34.2% of the calves, might be accepted as a paraphysiological deviation from complete anatomical compliance, the same cannot be said for the animals with partial dystopia and complete ectopia (6.1% and 2.1%, respectively), that should be considered as pathological deviations. In fact, the location and configuration of the SLAC significantly diverges from the normal where the centripetal and the centrifugal loops of the SLAC should be securely attached to the left side or the medial aspect of the mesojejunum. The physiological coil-shaped disposition of the loops attached to the mesojejunum is due to the movement to which the future coils of the ascending colon are subjected during embryonic development. According to the current understanding of intestinal development, the considerable elongation of the primitive intestine at approximately the 60^th^ day of the embryonic stage forces a part of the colon to wind itself as a flat wreath around a central bend (the future central flexure). An intestinal disk (the future SLAC) is formed with a more or less elliptical shape. Later in foetal development, this disc will superimpose itself on the left side of the mesojejunum. Thereafter, the ascending mesocolon will fuse with the mesojejunum integrating the vessels, and the fatty and the connective tissues definitely fasten the SLAC to the mesojejunum [2,10–12]. Consequently, in the physiological ontogenetically-developed animal, the SLAC is perfectly contained in the mesojejunum.

Results similar to those of the current study were obtained by Wölfl [9]; only 60.6% of the SLACs observed in the animals undergoing laparotomy or those which were examined post-mortem were considered normal. The degree of the anomalies observed was somehow also similar to that of the current study; 24.9% showed a slight anomaly (corresponding to the 34.2% conical shape looseness in this study), 9.8% showed moderate isolation from the mesentery disc (corresponding to the 6.1% partial dystopia in the current study) and 4.7% showed no embedding of the SLAC in the mesojejunum (corresponding to the 2.1% complete ectopia in the current study).

Unfortunately, in the current study, no information was available regarding the pedigree of the animals presenting partial dystopia or complete ectopia of the SLAC. However, given the high prevalence in the breed examined, the presence of some familial lines of Holstein cattle genetically carrying such anatomical variations cannot be excluded. Interestingly, with respect to another quite frequent congenital intestinal abnormality, atresia coli, Constable et al. [6] hypothesised regarding the presence of an allele representing a proportionately longer colon and/or rapid growth of the colon during the first trimester of pregnancy. This genetic asset would predispose calves to atresia coli due to a major susceptibility of damaging the intestinal vasculature during the developmental phase of the colon, leading to gut ischemia and atretic segments. Amniotic vecicle palpation between the 32^nd^ and the 40^th^ day of gestation (when the developing colon is in the umbilical cord) would place added traumatic stress on the colonic vasculature as it migrates together with the developing ascending and spiral colon. A similar mechanism could be hypothesised for the heterotopy of the SLAC. In this sense, the most delicate period might be the second and the third gestational months. In fact, the first evidence of looping of the proximal colon begins by day 45 still in the umbilical cord whereas extensive spiraling of the colon and its associated blood supply begins at day 56 of gestation, being completed by day 112. At this gestational time, the development of the bovine colon can be considered complete [6, 10].

The heterotopy of the SLAC resembles a developmental abnormality described in human medicine called “intestinal malrotation or nonrotation” [13–15]. Its incidence, estimated at between 0.2 and 1% [16–18], actually depends on whether it includes symptomatic patients only or also cases found incidentally by radiology or autopsy [19]. The term itself does not refer to a single congenital anomaly, but to a spectrum of developmental disorders which occurs when the normal embryological rotation and fixation of the intestine fail. Therefore, the relative position of other organs, such as the caecum, intestine and Meckel’s diverticulum, changes. Three clinical scenarios are considered in human medicine as a consequence of intestinal malrotation: a) patient with abdominal symptoms from malrotation, b) patient discovered to have malrotation during the evaluation and treatment of an abdominal complaint and c) asymptomatic patient with an incidental finding of malrotation at radiology or autopsy [19, 20]. Although children or adult with intestinal malrotation may remain asymptomatic for their entire life [21], some authors advocate preventive surgery (Ladd procedure) based on radiological diagnosis, due to the risk of acute and unexpected midgut volvulus as a consequence of intestinal malrotation [22, 23]. Significant sequelae of malrotation can occur despite years of symptom-free existence, although the timing of these sequelae cannot be predicted [16]. Interestingly, retrospective investigations have shown that not all asymptomatic patients were truly without symptoms; in fact, if questioned thoroughly, some of them actually affirmed having had some abdominal complaints which could be attributed to malrotation [16]. The exact cause of intestinal malrotation in human has not been determined; however, a familial factor was suspected [24].

If compared with the malrotation of the human medicine, the observations in the present study elicit important considerations. The slaughtered calves with heterotopy of the SLAC, especially those with partial dystopia or complete ectopia, could be considered asymptomatic patients with an incidental finding of “malrotation” post-mortem. In a previous study, Gentile et al. [25] found heterotopy of the SLAC in 32 out of 122 (26.2%) ascending colons of calves examined under 6 months of age which had died or had been euthanised for different reasons. Two of these calves suffered from intestinal torsion and chronic indigestion, suggesting a potential role of the heterotopy in the pathogenesis of the diseases. In that study, not only Holstein calves were represented.

In 2008, Rademacher and Gentile [8] correlated six different ileus conditions from intestinal obstruction to heterotopy of the SLAC, hypothesising the causative/predisposing pathogenetic role of the dysmorphism. Wölfl [9] observed an accumulation of clinically manifest caecal illnesses in connection with the defective integration of the SLAC into the mesojejunum: in 13 patients with clinical evidence of caecum disease only two of the SLACs corresponded to the anatomical morphology. In two animals, a bowel obstruction was caused by a *dilatatio and torsio caeci* around the non-integrated SLAC. An additional case of bowel obstruction was caused by the twisting of the stemlike and elongated jejunal mesentery around its own axis at the level of the non-integrated, cone-shaped SLAC.

**Fig 5:**
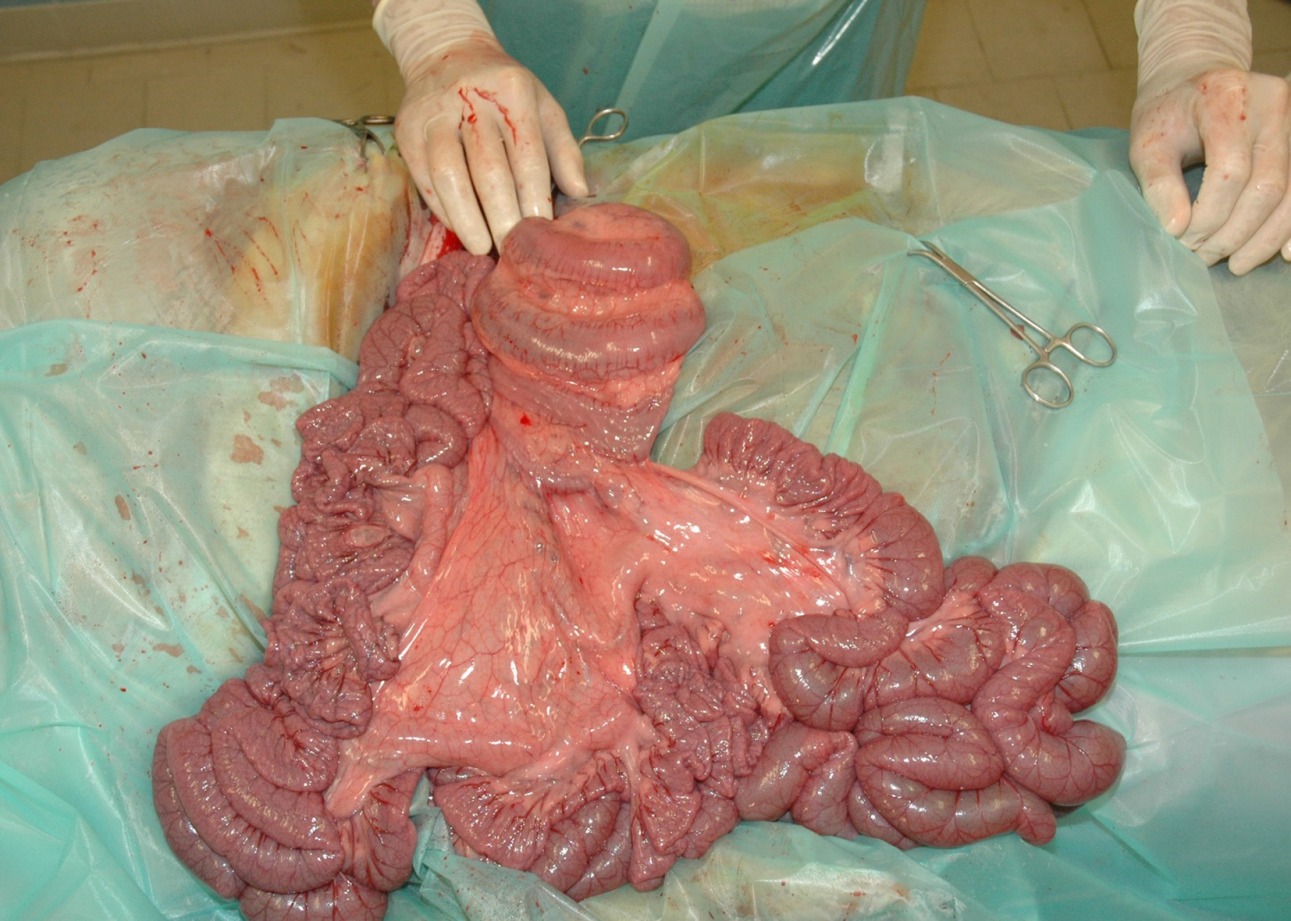
Intraoperative view from a right flank approach on a calf presented for recurrent colic. Notice the ectopic position and the abnormal coiling of the SLAC.

It could be hypothesised that the lack of a broad attachment of the SLAC to the mesentery (Fig 5) might provoke an alteration of the intestinal centre of gravity, enhancing the already asymmetrical distribution of weight between the jejunum and the descending colon. In fact, the most voluminous parts of the intestine, i.e. the caecum and the colon, are located within the supraomental recess caudodorsally to the small intestine [26]. An additional unbalancing caused by the partial or complete detachment of the SLAC would foster the risk of cranial-ventral slipping of the intestinal posterior tracts around an oblique caudoventrally oriented axis whereas the remaining part of the intestine, represented prevalently by smaller and therefore lighter loops, would be pushed caudodorsally. The entire situation would cause a rotational movement around a single fixed point which is the mesentery root. Moreover, abnormal relationships between the distinct intestinal tracts could compromise the proper functioning of the peristaltic motions, with the abnormal accumulation of gas and/or liquids within the loops whose consequent distension could constitutes additional upset.

Additional evidence of the potential instability of the intestine in maintaining its correct anatomical position when subjected to stress causing abnormal movements of the intestinal mass is the complete ectopia found in a calf which died as a result of a torsion of the root of the mesentery following the conservative treatment of a left displaced abomasum by rolling (A. Gentile, personal observation). Furthermore, the heterotopy of the SLAC and consequent looseness, together with increased intestinal mobility, may eventually predispose colonic intussusceptions by facilitating the advancement of the intussusceptum into the intussuscipiens; in particular, this hypothesis could be proposed with regard to the rare reports of invagination of the central flexure of the spiral loop [27] or other tracts of the spiral colon [6,7,28,29].

## Conclusions

This study described four different configurations of the SLAC in a population of 6-8-month-old calves, involving loose attachment of the spiral loops to the mesojejunum, abnormal elongation of the mesocolon, overlapping of adjacent loops, abnormal coiling and finally complete detachment of the SLAC from the mesojejunum.

It has been suggested that conical shape looseness should be considered a variation of the SLAC normally described in the literature regarding veterinary anatomy. However, based on this study and the authors’ observations in clinical cases, it has been hypothesised that partial dystopia and ectopia should be considered abnormal. This anatomic abnormaly can predispose the affected animal to intestinal injury, such as volvulus and intussusception, either directly to the affected segment or by weight discrepencies with adjacent parts and abnormal movements. Although the experience of human medicine with so-called intestinal malrotation addresses the pathogenetic hypothesis of the heterotopies of the SLAC toward a congenital developmental abnormality, at present, this hypothesis requires additional study.

Although the authors have observed a case of conical shape looseness in a goat died to dystocia, no studies in ruminants different from cattle are known.
